# Poly[[di-μ-aqua-(μ-4-formyl-2-meth­oxy­phenol­ato)disodium] 4-formyl-2-meth­oxy­phenolate]

**DOI:** 10.1107/S1600536810000711

**Published:** 2010-01-13

**Authors:** Muhammad Nadeem Asghar, Onur Şahin, Muhammad Nadeem Arshad, Uzma Mazhar, Islam Ullah Khan, Orhan Büyükgüngör

**Affiliations:** a Materials Chemistry Laboratory, Department of Chemistry, GC University, Lahore 54000, Pakistan; b Forman Christian College (Chartered University), Ferozepur Road, Lahore, Pakistan; c Department of Physics, Ondokuz Mayıs University, TR-55139 Samsun, Turkey

## Abstract

In the title coordination polymer, {[Na_2_(C_8_H_7_O_3_)(H_2_O)_4_](C_8_H_7_O_3_)}_*n*_, all the non-H atoms except the water O atoms lie on a crystallographic mirror plane. One sodium cation is bonded to four water O atoms and one vanillinate O atom in a distorted square-based pyramidal arrangement; the other Na^+^ ion is six-coordinated by four water O atoms and two vanillinate O atoms in an irregular geometry. One of the vanillinate anions is directly bonded to two sodium ions, whilst the other only inter­acts with the polymeric network by way of hydrogen bonds. In the crystal, a two-dimensional polymeric array is formed; this is reinforced by O—H⋯O hydrogen bonds, which generate *R*_2_^1^(6) and *R*_2_^2^(20) loops.

## Related literature

For related crystal structures, see: Velavan *et al.* (1995 ▶); Iwasaki (1973 ▶); Iwasaki *et al.* (1976 ▶); Usman *et al.* (2002 ▶); Li *et al.* (1999 ▶); Kaduk (2000 ▶). For graph-set notation, see: Bernstein *et al.* (1995 ▶).
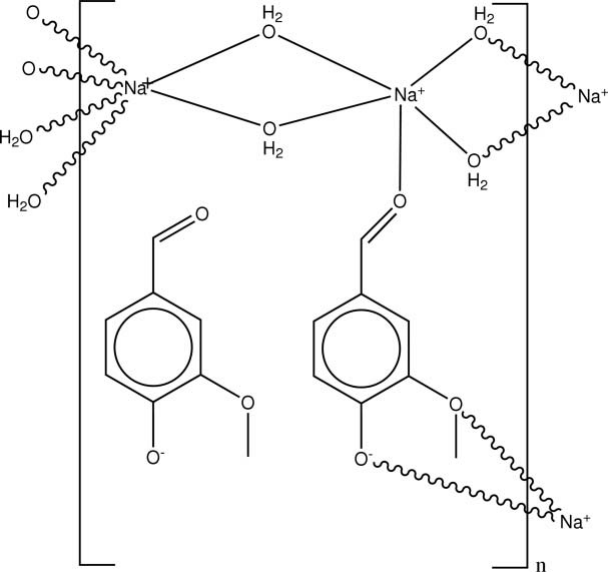


## Experimental

### 

#### Crystal data

[Na_2_(C_8_H_7_O_3_)(H_2_O)_4_](C_8_H_7_O_3_)
*M* *_r_* = 420.32
Orthorhombic, 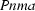

*a* = 12.2281 (6) Å
*b* = 6.7681 (3) Å
*c* = 22.6734 (10) Å
*V* = 1876.47 (15) Å^3^
*Z* = 4
Mo *K*α radiation
μ = 0.16 mm^−1^
*T* = 296 K
0.42 × 0.33 × 0.24 mm

#### Data collection

Bruker APEXII CCD diffractometer
Absorption correction: multi-scan (*SADABS*; Bruker, 2007 ▶) *T*_min_ = 0.938, *T*_max_ = 0.962
10469 measured reflections
2121 independent reflections
1825 reflections with *I* > 2σ(*I*)
*R*_int_ = 0.020

#### Refinement

*R*[*F* ^2^ > 2σ(*F* ^2^)] = 0.044
*wR*(*F* ^2^) = 0.119
*S* = 1.07
2121 reflections
180 parameters
12 restraints
H atoms treated by a mixture of independent and constrained refinement
Δρ_max_ = 0.63 e Å^−3^
Δρ_min_ = −0.67 e Å^−3^

### 

Data collection: *APEX2* (Bruker, 2007 ▶); cell refinement: *SAINT* (Bruker, 2007 ▶); data reduction: *SAINT*; program(s) used to solve structure: *SHELXS97* (Sheldrick, 2008 ▶); program(s) used to refine structure: *SHELXL97* (Sheldrick, 2008 ▶); molecular graphics: *ORTEP-3* (Farrugia, 1997 ▶); software used to prepare material for publication: *WinGX* (Farrugia, 1999 ▶) and *SHELXL97*.

## Supplementary Material

Crystal structure: contains datablocks global, I. DOI: 10.1107/S1600536810000711/hb5277sup1.cif

Structure factors: contains datablocks I. DOI: 10.1107/S1600536810000711/hb5277Isup2.hkl

Additional supplementary materials:  crystallographic information; 3D view; checkCIF report

## Figures and Tables

**Table:** 

Na1—O1	2.3751 (18)
Na1—O2	2.4043 (18)
Na1—O6	2.339 (3)
Na2—O1	2.5938 (19)
Na2—O2	2.4462 (18)
Na2—O7^i^	2.396 (2)
Na2—O8^i^	2.397 (2)

**Table:** 

O7^i^—Na2—O1	93.66 (6)
O8^i^—Na2—O1	138.31 (5)
O2^ii^—Na2—O1	126.01 (7)
O2—Na2—O1	75.61 (5)
O1^ii^—Na2—O1	75.98 (8)

Symmetry codes: (i) 
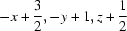
; (ii) 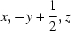.

**Table 2:** Hydrogen-bond geometry (Å, °)

*D*—H⋯*A*	*D*—H	H⋯*A*	*D*⋯*A*	*D*—H⋯*A*
O1—H1*A*⋯O5	0.82 (2)	1.97 (2)	2.772 (2)	169 (3)
O1—H1*B*⋯O8^iii^	0.83 (2)	1.99 (2)	2.815 (2)	172 (3)
O2—H2*A*⋯O4^iv^	0.82 (2)	2.07 (2)	2.872 (2)	168 (3)
O2—H2*B*⋯O5^v^	0.84 (2)	1.95 (2)	2.772 (2)	168 (3)

Symmetry codes: (iii) 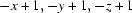; (iv) 
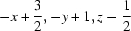
; (v) 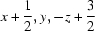.

**Fig. 1.**
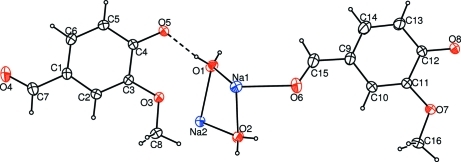
A view of the asymmetric unit of (I), showing displacement ellipsoids drawn at the 30% probability level. Hydrogen bonds are indicated by dashed lines.

**Fig. 2.**
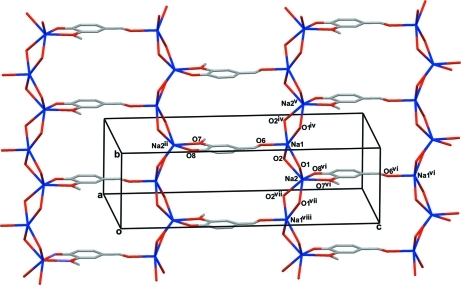
View of part of the crystal structure of compound (I), showing the formation of a coordination polymer chain parallel to the bc plane. For the sake of clarity, the noncoordinated molecule and all H atoms have been omitted. [Symmetry codes: (ii) 3/2 - x, 1 - y, z - 1/2; (iv) x, 3/2 - y, z; (v) x, 1 + y, z; (vi) 3/2 - x, 1 - y, 1/2 + z; (vii) x, 1/2 - y, z; (viii) x, y - 1, z.]

**Fig. 3.**
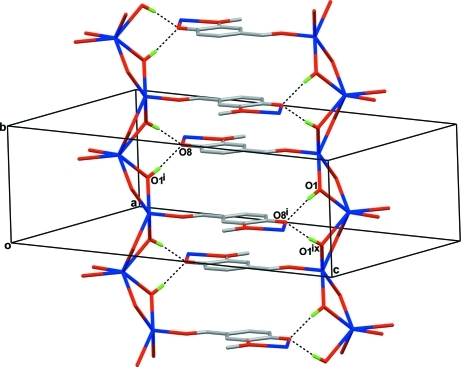
Part of the crystal structure of (I), showing the formation of R21(6) and R22(20) rings. For the sake of clarity, the noncoordinated molecule and H atoms have been omitted. [Symmetry codes: (i) 1 - x, 1 - y, 1 - z; (ix) x, 1/2 - y, z.]

**Fig. 4.**
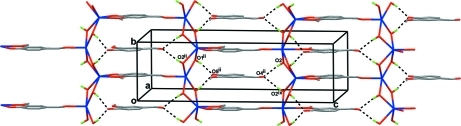
Part of the crystal structure of (I), showing the formation of R21(6) and R22(20) rings. H atoms not involved in these interactions have been omitted for clarity. [Symmetry codes: (ii) 3/2 - x, 1 - y, z - 1/2; (ix) x, 1/2 - y, z.]

## supplementary crystallographic information

### Comment

The crystal structures of vanillin-I (Velavan *et al.*, 1995), the polymorphic forms of isovanillin (Iwasaki, 1973), *o*-vanillin (Iwasaki *et al.*, 1976) and other vanillin derivatives (Usman *et al.*, 2002; Li *et al.*, 1999) have been reported.   We now report the title compound, (I).

The basic polymeric fragment of (I), with asymmetric unit formula [Na_2_(H_2_O)_2_(C_8_H_7_O_3_)_2_]_n_, is illustrated in Fig. 1. The Na^+^ cations are of two coordination types. In the first of these coordination, the Na1 coordination by four O atoms from two equivalent water molecules (O1, O2, O1^iv^ and O2^iv^) and the bonded carboxylate O atom from vanillin ligand (O6) (Table 1). In the second coordination, cation Na2 is coordinated by four O atoms from two equivalent water molecules (O1, O2, O1^vii^ and O2^vii^) and two O atoms from vanillin ligand (O7^vi^ and O8^vi^) [symmetry codes: (iv) *x*, 1/2 - *y*, *z*; (vi) 3/2 - *x*, -*y*, 1/2 + *z*; (vii) *x*, -1/2 - *y*, *z*]. The vanillin ligand five-membered (O7^vi^/C11^vi^/C12^vi^/O8^vi^/Na2) chelates to the Na atom through the methoxy and hydroxy groups. Two adjacent Na^+^ cations are linked together by two H_2_O bridges to form a four-membered ring with an Na_2_O_2_ core. The Na1···Na2 separation is 3.7595 (8) Å. Adjacent Na_2_O_2_ binuclear motifs are further joined by the vanillin ligand through carboxyl atoms O6, O7 and O8, to produce a one-dimensional chain along the *c* axis, with an Na1···Na2^ii^ separation of 9.890Å [symmetry code: (ii) 3/2 - *x*, 1 - *y*, *z* - 1/2]; this compares with the corresponding Na···Na distance of 8.006 (3)Å in the three-dimensional Na-terephthalate polymer [Na_2_(C_8_H_4_O_4_)] (Kaduk, 2000). These chains are connected by the water O atoms [Na1···Na2^v^ = 3.7595 (8) Å; symmetry code: (v) *x*, 1 + *y*, *z*], generating a two-dimensional layer architecture in the crystallographic *bc* plane (Fig. 2).

Water atom O1 in the molecule at (*x*, *y*, *z*) acts as a hydrogen-bond donor, *via* H1B, to atom O8^i^ so forming a C(10)[*R*_2_^2^(20)] (Bernstein *et al.*, 1995) chain of rings running parallel to the [0–10] direction and centrosymmetric *R*_2_^2^(20) rings centred at (1/2, 1/2+n/2, 1/2) (n = zero or integer). The combination of O1—H1B···O8^i^ and O1^ix^—H1B^ix^···O8^i^ hydrogen bonds produce *R*_2_^1^(6) ring (Fig. 3). Water atom O2 in the molecule at (*x*, *y*, *z*) acts as a hydrogen-bond donor, *via* H2A, to atom O4^ii^, while O1^ii^ acts as donor to O5^ii^, and in this manner a C_2_^2^(12) chain running parallel to the [00–1] direction. The combination of O2—H2A···O4^ii^, O1^ii^—H1A^ii^···O5^ii^ and O2^ix^—H2A^ix^···O4^ii^ hydrogen bonds produce *R*_2_^1^(6) and *R*_2_^2^(20) rings (Fig. 4).

### Experimental

Sodium hydroxide (0.66 g, 0.0165 mmol) was dissolved in a mixture of distilled water (10 ml) and ethanol (8 ml). The solution was cooled to room temperature. Half of the mixture of vanillin (1 g, 0.00658 mmol) and acetone (0.19 g, 0.00329 mmol) added to the above solution and stirred at room temperature for 15 minute then the remaining mixture was added and stirred for 2 h under the same conditions.  The greenish-yellow precipitate obtained was filtered and recystalized from methanol to yield colourless blocks of (I).

### Refinement

All H atoms bound to C atoms were refined using a riding model, with C—H = 0.93Å and *U*_iso_(H) = 1.2*U*_eq_(C) for aromatic C, and C—H = 0.96Å and *U*_iso_(H) = 1.5*U*_eq_(C) for methyl C atoms. Water H atom was located in difference maps and refined subject to a restraint of O—H = 0.83 (2) Å.

### Crystal data

**Table:**

[Na_2_(C_8_H_7_O_3_)(H_2_O)_4_](C_8_H_7_O_3_)	*F*(000) = 880
*M**_r_* = 420.32	*D*_x_ = 1.488 Mg m^−^^3^
Orthorhombic, *P**n**m**a*	Mo *K*α radiation, λ = 0.71073 Å
Hall symbol: -P 2ac 2n	Cell parameters from 5113 reflections
*a* = 12.2281 (6) Å	θ = 3.0–31.1°
*b* = 6.7681 (3) Å	µ = 0.16 mm^−^^1^
*c* = 22.6734 (10) Å	*T* = 296 K
*V* = 1876.47 (15) Å^3^	Block, colourles
*Z* = 4	0.42 × 0.33 × 0.24 mm

### Data collection

**Table:**

Bruker APEXII CCD diffractometer	2121 independent reflections
Radiation source: fine-focus sealed tube	1825 reflections with *I* > 2σ(*I*)
graphite	*R*_int_ = 0.020
phi and ω scans	θ_max_ = 26.5°, θ_min_ = 2.5°
Absorption correction: multi-scan (*SADABS*; Bruker, 2007)	*h* = −15→15
*T*_min_ = 0.938, *T*_max_ = 0.962	*k* = −8→4
10469 measured reflections	*l* = −19→28

### Refinement

**Table:**

Refinement on *F*^2^	Secondary atom site location: difference Fourier map
Least-squares matrix: full	Hydrogen site location: inferred from neighbouring sites
*R*[*F*^2^ > 2σ(*F*^2^)] = 0.044	H atoms treated by a mixture of independent and constrained refinement
*wR*(*F*^2^) = 0.119	*w* = 1/[σ^2^(*F*_o_^2^) + (0.0476*P*)^2^ + 1.7283*P*] where *P* = (*F*_o_^2^ + 2*F*_c_^2^)/3
*S* = 1.07	(Δ/σ)_max_ = 0.001
2121 reflections	Δρ_max_ = 0.63 e Å^−^^3^
180 parameters	Δρ_min_ = −0.67 e Å^−^^3^
12 restraints	Extinction correction: *SHELXL97* (Sheldrick, 2008), Fc^*^=kFc[1+0.001xFc^2^λ^3^/sin(2θ)]^-1/4^
Primary atom site location: structure-invariant direct methods	Extinction coefficient: 0.0138 (14)

### Special details

**Table:**

Geometry. All e.s.d.'s (except the e.s.d. in the dihedral angle between two l.s. planes) are estimated using the full covariance matrix. The cell e.s.d.'s are taken into account individually in the estimation of e.s.d.'s in distances, angles and torsion angles; correlations between e.s.d.'s in cell parameters are only used when they are defined by crystal symmetry. An approximate (isotropic) treatment of cell e.s.d.'s is used for estimating e.s.d.'s involving l.s. planes.
Refinement. Refinement of *F*^2^ against ALL reflections. The weighted *R*-factor *wR* and goodness of fit *S* are based on *F*^2^, conventional *R*-factors *R* are based on *F*, with *F* set to zero for negative *F*^2^. The threshold expression of *F*^2^ > σ(*F*^2^) is used only for calculating *R*-factors(gt) *etc*. and is not relevant to the choice of reflections for refinement. *R*-factors based on *F*^2^ are statistically about twice as large as those based on *F*, and *R*- factors based on ALL data will be even larger.

### Fractional atomic coordinates and isotropic or equivalent isotropic displacement parameters (Å^2^)

**Table:**

	*x*	*y*	*z*	*U*_iso_*/*U*_eq_	Occ. (<1)
C1	0.6494 (2)	0.7500	0.97772 (12)	0.0327 (7)	
C2	0.7164 (2)	0.7500	0.92725 (13)	0.0321 (7)	
H2	0.7920	0.7500	0.9315	0.039*	
C3	0.6713 (2)	0.7500	0.87213 (12)	0.0263 (6)	
C4	0.5554 (2)	0.7500	0.86488 (12)	0.0260 (6)	
C5	0.4908 (2)	0.7500	0.91595 (13)	0.0317 (7)	
H5	0.4151	0.7500	0.9123	0.038*	
C6	0.5368 (2)	0.7500	0.97117 (13)	0.0331 (7)	
H6	0.4921	0.7500	1.0044	0.040*	
C7	0.6978 (3)	0.7500	1.03533 (14)	0.0505 (10)	
H7	0.7738	0.7500	1.0370	0.061*	
C8	0.8444 (2)	0.7500	0.82464 (14)	0.0345 (7)	
H8A	0.8757	0.7214	0.7868	0.052*	0.50
H8B	0.8669	0.6512	0.8525	0.052*	0.50
H8C	0.8689	0.8774	0.8377	0.052*	0.50
C9	0.6086 (3)	0.7500	0.49146 (15)	0.0582 (12)	
C10	0.7012 (2)	0.7500	0.45577 (13)	0.0318 (7)	
H10	0.7704	0.7500	0.4728	0.038*	
C11	0.6907 (2)	0.7500	0.39567 (12)	0.0258 (6)	
C12	0.5862 (2)	0.7500	0.36734 (13)	0.0336 (7)	
C13	0.4958 (3)	0.7500	0.40446 (17)	0.092 (2)	
H13	0.4260	0.7500	0.3882	0.111*	
C14	0.5076 (3)	0.7500	0.46474 (18)	0.099 (2)	
H14	0.4452	0.7500	0.4882	0.118*	
C15	0.6143 (3)	0.7500	0.55467 (17)	0.0725 (15)	
H15	0.5477	0.7500	0.5745	0.087*	
C16	0.8841 (2)	0.7500	0.38079 (14)	0.0364 (7)	
H16A	0.8962	0.8710	0.4019	0.055*	0.50
H16B	0.9364	0.7389	0.3494	0.055*	0.50
H16C	0.8922	0.6401	0.4072	0.055*	0.50
O1	0.59561 (12)	0.4859 (3)	0.73010 (7)	0.0386 (4)	
H1A	0.5721 (19)	0.552 (4)	0.7574 (10)	0.047 (8)*	
H1B	0.5423 (18)	0.426 (4)	0.7162 (11)	0.060 (9)*	
O2	0.83431 (12)	0.4956 (3)	0.68524 (7)	0.0353 (4)	
H2A	0.835 (2)	0.439 (4)	0.6534 (9)	0.047 (8)*	
H2B	0.8935 (17)	0.557 (4)	0.6870 (11)	0.051 (8)*	
O3	0.72906 (16)	0.7500	0.82013 (9)	0.0352 (5)	
O4	0.6492 (2)	0.7500	1.08231 (10)	0.0528 (7)	
O5	0.51190 (16)	0.7500	0.81191 (8)	0.0319 (5)	
O6	0.6945 (2)	0.7500	0.58503 (11)	0.0611 (8)	
O7	0.77607 (16)	0.7500	0.35679 (9)	0.0367 (5)	
O8	0.57840 (16)	0.7500	0.31048 (8)	0.0325 (5)	
Na1	0.69720 (10)	0.7500	0.68819 (5)	0.0353 (3)	
Na2	0.75745 (10)	0.2500	0.75268 (5)	0.0343 (3)	

### Atomic displacement parameters (Å^2^)

**Table:**

	*U*^11^	*U*^22^	*U*^33^	*U*^12^	*U*^13^	*U*^23^
C1	0.0341 (15)	0.0405 (18)	0.0234 (14)	0.000	−0.0004 (11)	0.000
C2	0.0253 (13)	0.0429 (18)	0.0282 (14)	0.000	−0.0014 (11)	0.000
C3	0.0260 (13)	0.0284 (15)	0.0245 (13)	0.000	0.0031 (10)	0.000
C4	0.0265 (13)	0.0271 (15)	0.0245 (13)	0.000	−0.0018 (10)	0.000
C5	0.0241 (13)	0.0400 (17)	0.0309 (15)	0.000	0.0014 (11)	0.000
C6	0.0322 (14)	0.0404 (18)	0.0268 (14)	0.000	0.0053 (11)	0.000
C7	0.0400 (17)	0.084 (3)	0.0279 (16)	0.000	−0.0038 (13)	0.000
C8	0.0274 (14)	0.0409 (18)	0.0352 (16)	0.000	0.0065 (12)	0.000
C9	0.0366 (17)	0.110 (4)	0.0276 (17)	0.000	0.0020 (13)	0.000
C10	0.0299 (14)	0.0375 (17)	0.0281 (14)	0.000	−0.0045 (11)	0.000
C11	0.0268 (13)	0.0240 (14)	0.0267 (13)	0.000	0.0007 (10)	0.000
C12	0.0295 (14)	0.0457 (19)	0.0256 (14)	0.000	−0.0029 (11)	0.000
C13	0.0249 (16)	0.219 (7)	0.0333 (18)	0.000	−0.0012 (14)	0.000
C14	0.0368 (19)	0.218 (6)	0.041 (2)	0.000	0.0087 (17)	0.000
C15	0.047 (2)	0.138 (5)	0.0322 (19)	0.000	0.0065 (16)	0.000
C16	0.0245 (13)	0.0486 (19)	0.0363 (16)	0.000	−0.0056 (12)	0.000
O1	0.0325 (8)	0.0420 (10)	0.0413 (9)	−0.0063 (7)	0.0053 (7)	−0.0109 (8)
O2	0.0347 (8)	0.0402 (9)	0.0311 (8)	−0.0032 (7)	0.0039 (6)	−0.0014 (7)
O3	0.0256 (10)	0.0557 (15)	0.0243 (10)	0.000	0.0028 (8)	0.000
O4	0.0623 (16)	0.0727 (19)	0.0233 (11)	0.000	−0.0007 (10)	0.000
O5	0.0266 (9)	0.0442 (13)	0.0249 (10)	0.000	−0.0030 (8)	0.000
O6	0.0559 (16)	0.099 (2)	0.0287 (12)	0.000	−0.0051 (11)	0.000
O7	0.0243 (10)	0.0597 (15)	0.0262 (10)	0.000	−0.0024 (8)	0.000
O8	0.0291 (10)	0.0442 (13)	0.0242 (10)	0.000	−0.0039 (8)	0.000
Na1	0.0336 (6)	0.0352 (7)	0.0371 (7)	0.000	0.0020 (5)	0.000
Na2	0.0337 (6)	0.0422 (7)	0.0272 (6)	0.000	0.0001 (5)	0.000

### Geometric parameters (Å, °)

**Table:**

C1—C6	1.385 (4)	C12—C13	1.390 (5)
C1—C2	1.407 (4)	C13—C14	1.374 (6)
C1—C7	1.434 (4)	C13—H13	0.9300
C2—C3	1.366 (4)	C14—H14	0.9300
C2—H2	0.9300	C15—O6	1.198 (5)
C3—O3	1.374 (3)	C15—H15	0.9300
C3—C4	1.427 (4)	C16—O7	1.428 (3)
C4—O5	1.314 (3)	C16—H16A	0.9600
C4—C5	1.402 (4)	C16—H16B	0.9600
C5—C6	1.372 (4)	C16—H16C	0.9600
C5—H5	0.9300	O1—H1A	0.817 (17)
C6—H6	0.9300	O1—H1B	0.830 (17)
C7—O4	1.220 (4)	O2—H2A	0.816 (17)
C7—H7	0.9300	O2—H2B	0.836 (17)
C8—O3	1.414 (3)	Na1—O1	2.3751 (18)
C8—H8A	0.9600	Na1—O2	2.4043 (18)
C8—H8B	0.9600	Na1—O6	2.339 (3)
C8—H8C	0.9600	Na1—O1^i^	2.3751 (18)
C9—C14	1.376 (5)	Na1—O2^i^	2.4043 (18)
C9—C10	1.391 (4)	Na2—O1	2.5938 (19)
C9—C15	1.435 (5)	Na2—O2	2.4462 (18)
C10—C11	1.369 (4)	Na2—O2^ii^	2.4462 (18)
C10—H10	0.9300	Na2—O1^ii^	2.5938 (19)
C11—O7	1.366 (3)	Na2—O7^iii^	2.396 (2)
C11—C12	1.430 (4)	Na2—O8^iii^	2.397 (2)
C12—O8	1.293 (3)		
			
C6—C1—C2	119.4 (3)	H16A—C16—H16C	109.5
C6—C1—C7	120.6 (3)	H16B—C16—H16C	109.5
C2—C1—C7	120.0 (3)	Na1—O1—Na2	98.23 (6)
C3—C2—C1	120.6 (3)	Na1—O1—H1A	94.3 (19)
C3—C2—H2	119.7	Na2—O1—H1A	117.2 (19)
C1—C2—H2	119.7	Na1—O1—H1B	129 (2)
C2—C3—O3	125.3 (2)	Na2—O1—H1B	112 (2)
C2—C3—C4	120.4 (2)	H1A—O1—H1B	106 (2)
O3—C3—C4	114.3 (2)	Na1—O2—Na2	101.61 (6)
O5—C4—C5	121.8 (2)	Na1—O2—H2A	111.6 (19)
O5—C4—C3	120.5 (2)	Na2—O2—H2A	104.0 (19)
C5—C4—C3	117.7 (2)	Na1—O2—H2B	104.2 (19)
C6—C5—C4	121.5 (3)	Na2—O2—H2B	129.9 (19)
C6—C5—H5	119.2	H2A—O2—H2B	105 (2)
C4—C5—H5	119.2	C3—O3—C8	116.8 (2)
C5—C6—C1	120.3 (3)	C3—O3—Na1	141.66 (16)
C5—C6—H6	119.8	C8—O3—Na1	101.57 (16)
C1—C6—H6	119.8	C15—O6—Na1	125.9 (3)
O4—C7—C1	126.4 (3)	C11—O7—C16	117.4 (2)
O4—C7—H7	116.8	C11—O7—Na2^iv^	120.33 (16)
C1—C7—H7	116.8	C16—O7—Na2^iv^	122.24 (17)
O3—C8—H8A	109.5	C12—O8—Na2^iv^	118.88 (18)
O3—C8—H8B	109.5	O6—Na1—O1^i^	113.13 (7)
H8A—C8—H8B	109.5	O6—Na1—O1	113.13 (7)
O3—C8—H8C	109.5	O1^i^—Na1—O1	97.63 (9)
H8A—C8—H8C	109.5	O6—Na1—O2^i^	88.96 (7)
H8B—C8—H8C	109.5	O1^i^—Na1—O2^i^	80.61 (6)
C14—C9—C10	118.3 (3)	O1—Na1—O2^i^	156.19 (8)
C14—C9—C15	118.9 (4)	O6—Na1—O2	88.96 (7)
C10—C9—C15	122.8 (3)	O1^i^—Na1—O2	156.19 (8)
C11—C10—C9	120.2 (3)	O1—Na1—O2	80.61 (5)
C11—C10—H10	119.9	O2^i^—Na1—O2	91.48 (9)
C9—C10—H10	119.9	O6—Na1—O3	173.38 (9)
O7—C11—C10	124.8 (3)	O1^i^—Na1—O3	70.78 (5)
O7—C11—C12	113.1 (2)	O1—Na1—O3	70.78 (5)
C10—C11—C12	122.1 (3)	O2^i^—Na1—O3	86.42 (5)
O8—C12—C13	123.0 (3)	O2—Na1—O3	86.42 (5)
O8—C12—C11	120.9 (3)	O7^iii^—Na2—O8^iii^	66.71 (7)
C13—C12—C11	116.0 (3)	O7^iii^—Na2—O2^ii^	132.97 (5)
C14—C13—C12	121.3 (3)	O8^iii^—Na2—O2^ii^	91.15 (6)
C14—C13—H13	119.4	O7^iii^—Na2—O2	132.97 (5)
C12—C13—H13	119.4	O8^iii^—Na2—O2	91.15 (6)
C13—C14—C9	122.1 (4)	O2^ii^—Na2—O2	85.60 (9)
C13—C14—H14	118.9	O7^iii^—Na2—O1^ii^	93.66 (6)
C9—C14—H14	118.9	O8^iii^—Na2—O1^ii^	138.31 (5)
O6—C15—C9	127.8 (4)	O2^ii^—Na2—O1^ii^	75.61 (6)
O6—C15—H15	116.1	O2—Na2—O1^ii^	126.01 (7)
C9—C15—H15	116.1	O7^iii^—Na2—O1	93.66 (6)
O7—C16—H16A	109.5	O8^iii^—Na2—O1	138.31 (5)
O7—C16—H16B	109.5	O2^ii^—Na2—O1	126.01 (7)
H16A—C16—H16B	109.5	O2—Na2—O1	75.61 (5)
O7—C16—H16C	109.5	O1^ii^—Na2—O1	75.98 (8)
			
C6—C1—C2—C3	0.000 (2)	C10—C11—O7—Na2^iv^	180.0
C7—C1—C2—C3	180.000 (1)	C12—C11—O7—Na2^iv^	0.0
C1—C2—C3—O3	180.000 (1)	C13—C12—O8—Na2^iv^	180.0
C1—C2—C3—C4	0.000 (1)	C11—C12—O8—Na2^iv^	0.0
C2—C3—C4—O5	180.000 (1)	C15—O6—Na1—O1^i^	−54.92 (6)
O3—C3—C4—O5	0.000 (1)	C15—O6—Na1—O1	54.92 (6)
C2—C3—C4—C5	0.000 (1)	C15—O6—Na1—O2^i^	−134.25 (4)
O3—C3—C4—C5	180.000 (1)	C15—O6—Na1—O2	134.25 (4)
O5—C4—C5—C6	180.000 (1)	Na2—O1—Na1—O6	99.60 (9)
C3—C4—C5—C6	0.000 (2)	Na2—O1—Na1—O1^i^	−141.19 (5)
C4—C5—C6—C1	0.000 (2)	Na2—O1—Na1—O2^i^	−57.16 (18)
C2—C1—C6—C5	0.000 (2)	Na2—O1—Na1—O2	14.80 (6)
C7—C1—C6—C5	180.000 (1)	Na2—O1—Na1—O3	−74.67 (6)
C6—C1—C7—O4	0.000 (2)	Na2—O2—Na1—O6	−129.53 (7)
C2—C1—C7—O4	180.000 (2)	Na2—O2—Na1—O1^i^	71.75 (18)
C14—C9—C10—C11	0.0	Na2—O2—Na1—O1	−15.88 (7)
C15—C9—C10—C11	180.0	Na2—O2—Na1—O2^i^	141.54 (5)
C9—C10—C11—O7	180.0	Na2—O2—Na1—O3	55.22 (6)
C9—C10—C11—C12	0.0	C3—O3—Na1—O1^i^	52.85 (5)
O7—C11—C12—O8	0.0	C8—O3—Na1—O1^i^	−127.15 (5)
C10—C11—C12—O8	180.0	C3—O3—Na1—O1	−52.85 (5)
O7—C11—C12—C13	180.0	C8—O3—Na1—O1	127.15 (5)
C10—C11—C12—C13	0.0	C3—O3—Na1—O2^i^	134.14 (4)
O8—C12—C13—C14	180.0	C8—O3—Na1—O2^i^	−45.86 (4)
C11—C12—C13—C14	0.0	C3—O3—Na1—O2	−134.14 (4)
C12—C13—C14—C9	0.000 (1)	C8—O3—Na1—O2	45.86 (4)
C10—C9—C14—C13	0.000 (1)	Na1—O2—Na2—O7^iii^	−66.66 (11)
C15—C9—C14—C13	180.0	Na1—O2—Na2—O8^iii^	−125.21 (6)
C14—C9—C15—O6	180.0	Na1—O2—Na2—O2^ii^	143.73 (5)
C10—C9—C15—O6	0.000 (1)	Na1—O2—Na2—O1^ii^	75.08 (9)
C2—C3—O3—C8	0.000 (1)	Na1—O2—Na2—O1	14.79 (6)
C4—C3—O3—C8	180.000 (1)	Na1—O1—Na2—O7^iii^	118.71 (6)
C2—C3—O3—Na1	180.000 (1)	Na1—O1—Na2—O8^iii^	60.30 (12)
C4—C3—O3—Na1	0.000 (1)	Na1—O1—Na2—O2^ii^	−88.29 (9)
C9—C15—O6—Na1	180.0	Na1—O1—Na2—O2	−14.81 (6)
C10—C11—O7—C16	0.0	Na1—O1—Na2—O1^ii^	−148.42 (4)
C12—C11—O7—C16	180.0		

Symmetry codes: (i) *x*, −*y*+3/2, *z*; (ii) *x*, −*y*+1/2, *z*; (iii) −*x*+3/2, −*y*+1, *z*+1/2; (iv) −*x*+3/2, −*y*+1, *z*−1/2.

### Hydrogen-bond geometry (Å, °)

**Table:**

*D*—H···*A*	*D*—H	H···*A*	*D*···*A*	*D*—H···*A*
O1—H1A···O5	0.82 (2)	1.97 (2)	2.772 (2)	169 (3)
O1—H1B···O8^v^	0.83 (2)	1.99 (2)	2.815 (2)	172 (3)
O2—H2A···O4^iv^	0.82 (2)	2.07 (2)	2.872 (2)	168 (3)
O2—H2B···O5^vi^	0.84 (2)	1.95 (2)	2.772 (2)	168 (3)

Symmetry codes: (v) −*x*+1, −*y*+1, −*z*+1; (iv) −*x*+3/2, −*y*+1, *z*−1/2; (vi) *x*+1/2, *y*, −*z*+3/2.
